# Needle morphological evidence of the homoploid hybrid origin of *Pinus densata* based on analysis of artificial hybrids and the putative parents, *Pinus tabuliformis* and *Pinus yunnanensis*

**DOI:** 10.1002/ece3.1062

**Published:** 2014-04-19

**Authors:** Fangqian Xing, Jian-Feng Mao, Jingxiang Meng, Jianfeng Dai, Wei Zhao, Hao Liu, Zhen Xing, Hua Zhang, Xiao-Ru Wang, Yue Li

**Affiliations:** 1 National Engineering Laboratory for Forest Tree Breeding, Key Laboratory for Genetics and Breeding of Forest Trees and Ornamental Plants of Ministry of Education, Beijing Forestry UniversityBeijing, 100083, China; 2 State Key Laboratory of Systematic and Evolutionary Botany, Institute of Botany, Chinese Academy of SciencesBeijing, 100093, China; 3 College of Resources and Environment, College of agriculture and animal husbandry of Tibet UniversityLinzhi, 860000, China; 4 Department of Ecology and Environmental Science, Umeå UniversitySE-901 87, Umeå, Sweden

**Keywords:** Anatomy, artificial hybrid, morphology, *Pinus densata*, *Pinus tabuliformis*, *Pinus yunnanensis*

## Abstract

Genetic analyses indicate that *Pinus densata* is a natural homoploid hybrid originating from *Pinus tabuliformis* and *Pinus yunnanensis*. Needle morphological and anatomical features show relative species stability and can be used to identify coniferous species. Comparative analyses of these needle characteristics and phenotypic differences between the artificial hybrids, *P. densata*, and parental species can be used to determine the genetic and phenotypic evolutionary consequences of natural hybridization. Twelve artificial hybrid families, the two parental species, and *P. densata* were seeded in a high-altitude habitat in Linzhi, Tibet. The needles of artificial hybrids and the three pine species were collected, and 24 needle morphological and anatomical traits were analyzed. Based on these results, variations in 10 needle traits among artificial hybrid families and 22 traits among species and artificial hybrids were predicted and found to be under moderate genetic control. Nineteen needle traits in artificial hybrids were similar to those in *P. densata* and between the two parental species, *P. tabuliformis* and *P. yunnanensis*. The ratio of plants with three needle clusters in artificial hybrids was 22.92%, which was very similar to *P. densata*. The eight needle traits (needle length, the mean number of stomata in sections 2 mm in length of the convex and flat sides of the needle, mean stomatal density, mesophyll/vascular bundle area ratio, mesophyll/resin canal area ratio, mesophyll/(resin canals and vascular bundles) area ratio, vascular bundle/resin canal area ratio) relative to physiological adaptability were similar to the artificial hybrids and *P. densata*. The similar needle features between the artificial hybrids and *P. densata* could be used to verify the homoploid hybrid origin of *P. densata* and helps to better understand of the hybridization roles in adaptation and speciation in plants.

## Introduction

*Pinus densata* is a pine species with a high-elevation niche, ranging from 2700 to 4200 m above sea level (a.s.l.) (Wu 1956; Guan 1981) in the southeastern region of the Tibetan Plateau. Molecular genetic analysis has supported that *P. densata* originated through homoploid hybridization between *Pinus tabuliformis* and *Pinus yunnanensis* (Wang and Szmidt 1990, 1994; Wang et al. 2011). The distribution and altitudinal range of the three species forms a succession: *P. densata* is found at the highest altitude of all *Pinus* in Asia, while *P. yunnanensis* is found in a relatively limited range in southwestern China from 700 to 3000 m. *Pinus tabuliformis* is widely distributed from northern to central China at altitudes below 2700 m a.s.l. (Wu 1956; Zheng 1983). The putative ancestral hybrid zone of *P. densata* was thought to be in the southwest of Sichuan Province, within the natural conjunction region between the two hypothetical parent species. *P. densata* experienced strong population differentiation during evolution, after which it formed its contemporary population (Wang et al. 2011; Gao et al. 2012).

Evidence of successful hybridization between the two parental species and a comparison of needle traits between the artificial hybrids and *P. densata* would increase our understanding of homoploid hybridization speciation. Although the parents of many existing hybrid species are unknown or extinct, hybrids obtained through artificial hybridization between hypothetical parental species could simulate the ancestral genotype of hybrid species (Gross and Rieseberg 2005). Morphological traits of a natural hybrid are the result of environmental characteristics, genetic diversity, and cooperative long-term selection and evolution (Rieseberg et al. 1999; Donovan et al. 2010). Despite the differences between artificial and natural hybrids, artificial hybrids can still provide important scientific insight into the evolution of early formed natural hybrids. Morphological and anatomical features of coniferous needle leaves can be used as references for the taxonomic identification of pine species because they have a certain degree of stability within species and are significantly different between species (Boratynska and Bobowicz 2001). We performed artificial hybridization between *P. tabuliformis* and *P. yunnanensis*, collected the artificial hybrid seeds, and established a field experiment of seedlings from the artificial hybrids and samples of *P. densata*, as well as the two parental species. Needles from the third-year seedlings were sampled to evaluate the genetic variation regulating needle morphological and anatomical traits among artificial hybrid families; understand the similarities and variations in needle morphological and anatomical traits across artificial hybrids, *P. densata*, *P. tabuliformis*, and *P. yunnanensis* and establish relationships between structural characteristics of needles and species adaptability. In this study, we compared needle trait variations in artificial hybrids and *P. densata* to examine similarities and differences with parental species from an adaptive and evolutionary perspective. The results provide morphological information for helping to understand the evolution of *P. densata*.

## Materials and Methods

### Artificial hybridization and species sampling

**Figure 1:**
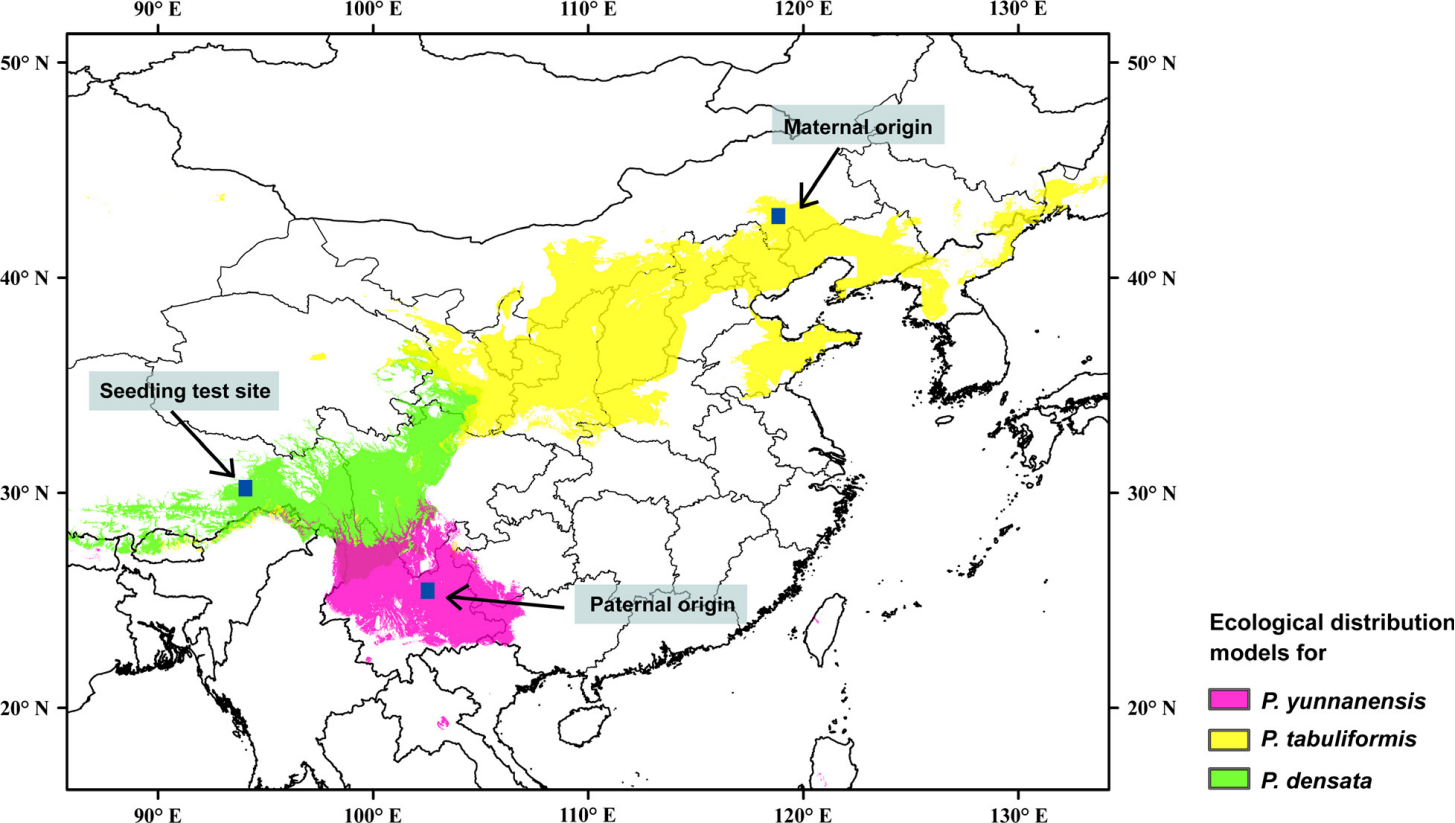
Test sites and ecological distribution models for *Pinus densata* and its theoretical parental species.

The artificial hybridization experiment was conducted in a seed orchard of *P. tabuliformis* in Ningcheng, Inner Mongolia, and six clones of *P. tabuliformis* were selected as the female parents. Pollen was collected from five individuals of *P. yunnanensis* in Kunming, Yunnan Province, as male parents. Artificial hybridization between the two species was carefully performed by bagging female flowers, performing pollination, preserving conelets, and collecting mature cones. Twelve hybrid families (Table 1) had sufficient full-seeds for field comparison trials. Open-pollinated seeds from the six female clones were used as *P. tabuliformis* (Pt) samples, mixed seeds from the five pollen trees were used as *P. yunnanensis* (Py) samples, and mixed seeds from *P. densata* populations in Linzhi, Tibet, were used as *P. densata* (Pd) samples. Representing each parental species with mixed parent tree open-pollinated seeds can be used to compare variations between hybrids and parental species. As the sampling population of each pine species was within the natural distribution region (Fig. 1) and no other pine species forests were located nearby, hybridization between species should not be possible. We also performed reciprocal hybridization with *P. yunnanensis* as the maternal parent and *P. tabuliformis* as the pollen parent using the same method and seeds as discussed above.

**Table 1:** Twelve artificial hybrid families between the maternal parent *Pinus tabuliformis* and the paternal parent *Pinus yunnanensis*

Artificial hybrid families	Cross combinations	Artificial hybrid families	Cross combinations
A	Pt6 × Py2	G	Pt5 × Py1
B	Pt6 × Py1	H	Pt1 × Py1
C	Pt2 × Py3	I	Pt4 × Py4
D	Pt3 × Py4	J	Pt1 × Py3
E	Pt3 × Py3	K	Pt1 × Py4
F	Pt3 × Py5	L	Pt2 × Py4

Six clones (Pt1–Pt6) of *P. tabuliformis* were incorporated as maternal parent, and five genotypes (Py1–Py5) of *P. yunnanensis* were included as paternal parent.

### Study site and field experiment

The location of the seedling field test was in Linzhi, Tibet (96.5°N, 94.36°E; 2950 m a.s.l.), which represents the distribution center of *P. densata*. The area is a plateau semi-humid monsoon climate zone, with an annual minimum temperature of −5.3°C, maximum temperature of 22.1°C, and annual average temperature of 8.5°C. The annual precipitation is 650 mm. Soil of the test field was sandy loam, >60 cm thick, with low–intermediate fertility (seedlings of *P. densata* grow well in the field).

### Field test experimental design

The experimental design of the field test was a randomized complete block (RCB), with 10 plants in each hybrid family (HF) plot and 20 plants in each species plot. Three block replications were performed. Field planting spacing and seedling management were the same for all plots.

### Needle sample collection

Needle samples were collected in October of the third year of plant growing. Ten to 15 seedlings were randomly selected from each HF or species. The 10 longest needles of each selected plant were collected, and three needles were chosen randomly for morphological and anatomical needle trait measurements. Ten to 15 individuals and 30–45 needles for each HF or species were analyzed.

### Morphological needle traits

The number of needles in a cluster for each parent species and *P. densata* differed. The majority of plants in *P. tabuliformis* contained two needles in a cluster, while *P. yunnanensis* contained three and *P. densata* contained two or three. We counted the ratio of plants with three needle clusters for each species and the artificial hybrids, measured a total of 20 morphological and anatomical needle traits, and derived four area ratio traits (comparative traits between needle organizational structures in needle cross sections).

**Figure 2:**
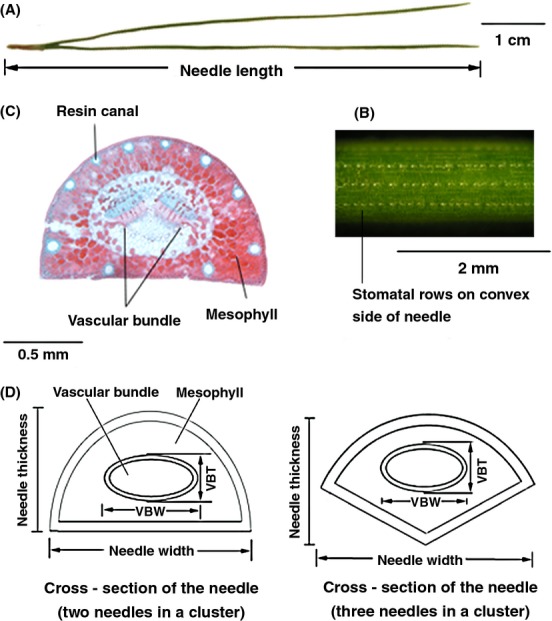
(A) needle length, (B) cross-section of the needle by paraffin section, (C) stomatal rows on convex side of needle, (D) cross-section of the needle (two needles in a cluster and three needles in a cluster).

The needle morphological traits (Fig. 2A and B) included needle length (NL, 1 mm), width (NW, 0.1 mm), and thickness (NT, 0.1 mm) at the middle of the needle. The number of stomatal rows on the convex and flat sides of the needle (CSRN and FSRN, respectively) were measured under a stereomicroscope, and the mean number of stomata in sections 2 mm in length of the convex and flat sides of the needle (CSR2N and FSR2N, respectively) were counted using Photoshop CS5 (Adobe Systems, Mountain View, CA) after photographing.

The stomatal density of the convex and flat sides of the needle were calculated (CSD and FSD, respectively), and the mean stomatal density (MSD) was determined (Zhao et al. 2008) as CSD = CSRN × CSR2N/(2 × NW); FSD = FSRN × FSR2N/(2 × NW); MSD = (CSD + FSD)/2.

### Anatomical needle traits

Slicing by hand (Xu and Tao 2006) was used to cut the middle of each needle, which was then observed under a microscope (BA2100; Motic, Xiamen, China). The needle tissue and structures of the vascular bundles, resin canals, and mesophyll, which function in substance transduction, stress tolerance, and organic synthesis, respectively, were measured.

Data on each anatomical needle trait (Fig. 2C and D) were determined using Motic Images Plus 2.0 software, as follows: vascular bundle width (VBW, 0.001 mm), vascular bundle thickness (VBT, 0.001 mm), vascular bundle area (VBA, 0.001 mm^2^), vascular bundle perimeter (VBP, 0.001 mm), resin canal number (RCN), total resin canal area (RCA, 0.001 mm^2^), total resin canal perimeter (RCP, 0.001 mm), needle section area (NSA, 0.01 mm^2^), needle section perimeter (NSP, 0.001 mm), and mesophyll area (MA, 0.01 mm^2^) (NSA − VBA − RCA).

### Area ratio traits between different organizational structures in needle cross sections

The area ratio traits between different organizational structures in needle cross sections include the mesophyll/vascular bundle area ratio (MA/VBA), mesophyll/resin canal area ratio (MA/RCA), mesophyll/(resin canals and vascular bundles) area ratio [MA/(VBA + RCA)], and vascular bundle/resin canal area ratio (VBA/RCA).

The mesophyll/VBA ratio represents a comparison of needle organic synthesis to water and inorganic salt transduction; the mesophyll/RCA ratio represents a comparison of needle organic synthesis and transduction, the mesophyll/(resin canals and vascular bundles) area ratio represents a comparison of needle organic synthesis to water and inorganic and organic transduction, and the vascular bundle/RCA ratio represents a comparison of needle water and inorganic transduction and organic synthesis (Table 2).

**Table 2:** Needle morphological and anatomical traits analyzed

Abbreviation	Unit	Traits
NL	mm	Needle length
NW	mm	Needle width
NT	mm	Needle thickness
CSRN	No.	Number of stomatal rows on convex side of needle
FSRN	No.	Number of stomatal rows on flat side of needle
CSR2N	No.	Mean number of stomata in a 2-mm-long section of convex side of needle
FSR2N	No.	Mean number of stomata in a 2-mm-long section of flat side of needle
CSD	No./mm^2^	Stomatal density on the convex surface of needle
FSD	No./mm^2^	Stomatal density on the flat surface of needle
MSD	No./mm^2^	Mean stomatal density of needle
VBW	mm	Vascular bundle width
VBT	mm	Vascular bundle thickness
NSA	mm^2^	Needle section area
NSP	mm	Needle section perimeter
VBA	mm^2^	Vascular bundle area
VBP	mm	Vascular bundle perimeter
RCN	No.	Resin canal number
RCA	mm^2^	Resin canals area
RCP	mm	Resin canals perimeter
MA	mm^2^	Mesophyll area
MA/VBA	–	Mesophyll area/vascular bundle area
MA/RCA	–	Mesophyll area/resin canals area
MA/(VBA + RCA)	–	Mesophyll area/(resin canals area and vascular bundle area)
VBA/RCA	–	Vascular bundle area/resin canals area

### Statistical analysis

Means, standard deviations, and the coefficients of variation (CVs) for each trait among hybrid families, maternal species, paternal species, and *P. densata* were estimated. Analyses of variance (ANOVAs) of each morphological and anatomical needle trait among artificial hybrids and pine species, as well as hybrid families, were analyzed using a similar nested linear model and estimates of variance:


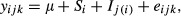


where *y*_*ijk*_ is the sample needle value; *μ* is the experimental mean; *S*_*i*_ is the effect of species (fixed), artificial hybrids (fixed), or hybrid family (HF, random); *I*_*j(i)*_ is the effect (random) of individuals within species, artificial hybrids, or HF; and *e*_*ijk*_ is the error among sample needles (random). The significance of differences between artificial hybrids and pine species was examined using Duncan's multiple range test.

Clustering analysis was based on the relationship between needle morphological and anatomical traits of 12 hybrid families and three other species based on Euclidean distance. Nested ANOVA among species and hybrid families was performed in SAS (SAS Institute Inc., Raleigh, NC), and principal components analysis (PCA) of individuals within each species and artificial hybrids was performed in R (R, The University of Auckland, Auckland, New Zealand). In all analyses, *P* < 0.05 was considered to indicate statistical significance. Other analyses were performed using SPSS19.0 software (SPSS Inc., Chicago, IL).

The hybrid family heritability (H^2^) of each trait was calculated as described by Becker (1992). The individual repeatability (R) of each trait was estimated according to Falconer and Mackay (1996): 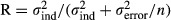, where 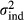 is the variance for individual and 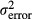 is the residual variance.

## Results

### Variations in morphological and anatomical needle traits among hybrid families

The ratio of the number of three needle clusters for 12 hybrid families ranged from 0% to 76.92%. Among them, the ratios in four hybrid families were <10%, the ratios in two hybrid families were between 10% and 20%, and the ratios in six hybrid families were >30%.

Descriptive statistics and CVs of all measured needle traits computed for 12 artificial hybrid families are provided in Table 3. All needle traits of hybrid families showed different variation ranges, and the variation ranges of NL, RCA, and MA/RCA nearly doubled. The CVs for the 24 needle traits among the 12 artificial hybrid families were within 10.26–55.55%. The CVs of FSR2N, CSR2N, and NSP were the smallest (CV < 12%), while those of RCA, RCP, RCN, MA/RCA, and VBA/RCA were the largest (CV > 37%). These results indicated that needle traits showed different degrees of phenotypic differentiation among the 12 artificial hybrid families.

**Table 3:** Statistics of each measured needle trait among 12 artificial hybrid families

Traits (unit)	Mean	SD^1^	CV%^2^	Min–Max	Traits (unit)	Mean	SD	CV%	Min–Max
NL (mm)	89.74	27.51	30.65	64.27–126.1	NSA (mm^2^)	0.59	0.145	24.13	0.50–0.67
NW (mm)	1.16	0.16	13.52	1.08–1.24	NSP (mm)	3.07	0.37	11.94	2.83–3.27
NT (mm)	0.68	0.11	15.60	0.62–0.76	VBA (mm^2^)	0.13	0.04	28.79	0.10–0.14
CSRN (No.)	6.56	1.57	23.90	5.76–8.25	VBP (mm)	1.33	0.19	14.48	1.19–1.45
FSRN (No.)	5.65	1.13	20.00	4.82–6.67	RCN (No.)	3.58	1.61	45.13	2.63–4.45
CSR2N (No.)	20.91	2.22	10.64	19.7–22.09	RCA (mm^2^)	0.002	0.001	55.55	0.001–0.002
FSR2N (No.)	20.46	2.10	10.26	19.22–21.75	RCP (mm)	0.31	0.15	49.22	0.23–0.38
CSD (No./mm^2^)	59.95	16.31	27.21	51.21–83.37	MA (mm^2^)	0.46	0.11	23.72	0.40–0.53
FSD (No./mm^2^)	50.20	11.15	22.22	43.71–61.39	MA/VBA	3.75	0.57	15.14	3.27–4.22
MSD (No./mm^2^)	55.07	12.55	22.79	47.46–72.38	MA/RCA	326.7	122.5	37.50	245.2–425.8
VBW (mm)	0.50	0.08	15.00	0.44–0.55	MA/(VBA + RCA)	3.70	0.56	15.06	3.23–4.15
VBT (mm)	0.30	0.05	15.78	0.27–0.34	VBA/RCA	89.12	33.28	37.35	63.3–132.4

CV, coefficients of variation; MSD, mean stomatal density; NL, needle length; NSA, needle section area; NSP, needle section perimeter; NT, needle thickness; NW, needle width; RCA, resin canal area; RCN, resin canal number; RCP, resin canal perimeter; VBA, vascular bundle area; VBP, vascular bundle perimeter; VBT, vascular bundle thickness; VBW, vascular bundle width.

1 Standard deviation.

2 Variance coefficient.

The results of ANOVA for 24 needle traits among hybrid families are shown in Table 4. Significant variation among hybrid families was observed based on 10 needle traits, while variations for traits such as NL, MA/VBA, MA/(VBA + RCA), and VBA/RCA were highest (*P* < 0.01). The variance components of the four traits were 26.06%, 12.99%, 12.27%, and 9.87% among hybrid families, respectively, and variance components for the other six traits with significant variation ranged from 2.40% to 7.58%. A relatively high HF broad sense heritability was estimated for NL, MA/VBA, MA/(VBA + RCA), and VBA/RCA (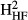 of 0.80, 0.68, 0.67, and 0.65, respectively), while heritability for the other six traits ranged from 0.34 to 0.52. Traits with moderate genetic control may experience microevolution within hybrid families. Significant variation (*P* < 0.05) was detected among individuals within the HF in all needle traits, and variance components of the needle traits ranged from 30.17% to 80.81%. The repeatability (R) estimated for all needle traits ranged from 0.57 to 0.99. Highly significant variation between individuals within the HF suggests that the needle traits were more stable among measurements within an individual but differed significantly among individuals.

**Table 4:** ANOVA of 24 needle traits among artificial hybrid families

Traits^1^	Mean square			Variance component (%)			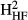	R_ind_
	
	HF^2^	Ind (HF)^3^	Error^4^	HF	Ind (HF)	Error		
NL	10945.47**	2140.42**	22.95	26.06	71.61	2.33	0.80	0.99
NW	0.062	0.065**	0.011	0.00	62.56	37.44	0.00	0.83
NT	0.060*	0.031**	0.005	5.92	60.47	33.61	0.48	0.84
CSRN	8.229*	5.423**	1.247	3.01	51.16	45.83	0.34	0.77
FSRN	8.216*	3.435**	0.835	7.58	47.08	45.34	0.58	0.76
CSR2N	18.319*	10.861**	3.619	3.48	38.62	57.90	0.41	0.67
FSR2N	12.962*	8.522**	3.638	2.40	30.17	67.43	0.34	0.57
CSD	668.585	562.497**	160.555	1.04	45.02	53.94	0.16	0.71
FSD	681.012*	330.228**	102.413	5.43	40.27	54.31	0.52	0.69
MSD	544.030	368.858**	80.312	2.81	52.96	44.22	0.32	0.78
VBW	0.017	0.016**	0.003	0.25	60.65	39.10	0.06	0.82
VBT	0.010	0.007**	0.001	3.35	67.02	29.62	0.30	0.87
NSA	0.070	0.069**	0.005	0.08	80.81	19.11	0.01	0.93
NSP	0.445	0.417**	0.038	0.49	76.63	22.88	0.06	0.91
VBA	0.004	0.004**	0.000	0.00	78.59	21.41	0.00	0.92
VBP	0.103	0.105**	0.014	0.00	68.36	31.64	0.00	0.87
RCN	6.849	6.922**	1.173	0.00	62.04	37.96	0.01	0.83
RCA	0.0048	0.0041**	0.0004	1.28	75.37	23.36	0.15	0.91
RCP	0.074	0.072**	0.008	0.19	71.12	28.69	0.03	0.88
MA	0.049	0.041**	0.003	1.49	79.54	18.97	0.16	0.93
MA/VBA	2.808**	0.893**	0.114	12.99	60.39	26.62	0.68	0.87
MA/RCA	56447.95	42909.190**	8709.951	1.93	55.60	42.48	0.24	0.80
MA/(VBA + RCA)	2.568**	0.853**	0.110	12.27	60.81	26.92	0.67	0.87
VBA/RCA	9053.082**	3182.353**	754.271	9.87	46.65	43.48	0.65	0.76

HF is hybrid family effect; ind (HF) is effect of individuals within hybrid family; *P < 0.05; **P < 0.01 for all variables in hybrid families (HF) and ind (HF).

1 See Table 2 for definitions of the variables.

2 df = 11 for hybrid family.

3 df = 127 for individuals within hybrid family.

4 df = 278 for error.

Compared with the effect of hybrid families, variance components had higher values in individuals within the HF. As trees in pine species are typically heterozygotes (Wang et al. 2001), the larger genetic variation of needle traits among individuals within the HF is due to both the parental species effect and parental tree effect and establish the evolutionary basis of *P. densata* in a unique hybrid niche.

### Comparison between artificial hybrids and three pine species

**Figure 3:**
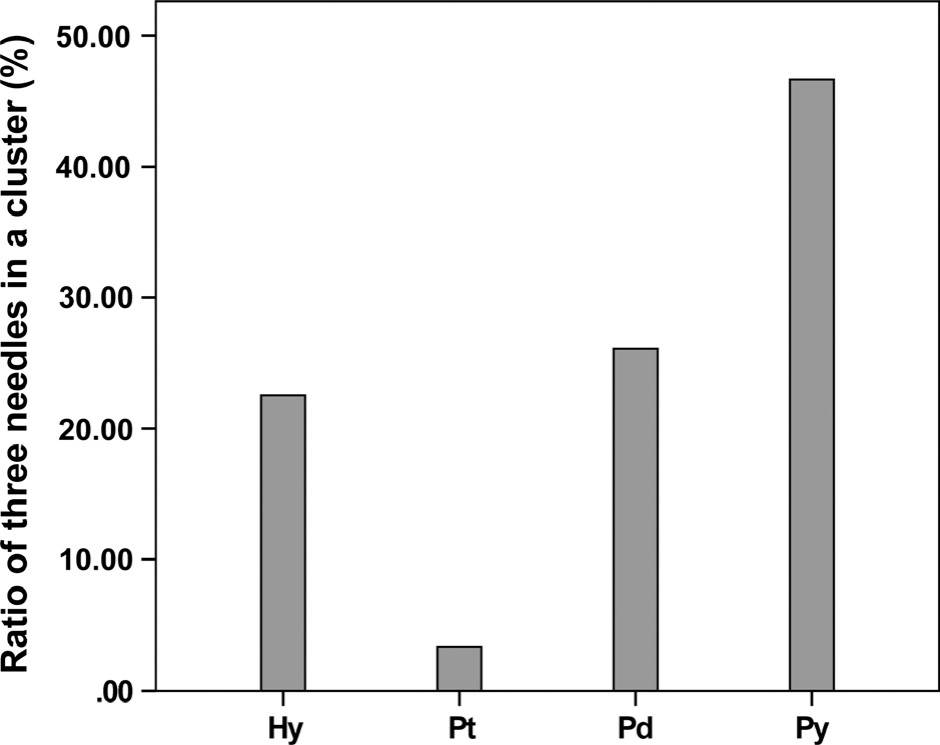
Ratios of three needle clusters of artificial hybrids and another three species. Hy: artificial hybrid, Pt: *Pinus tabuliformis*, Pd: *P. densata* and Py: *P. yunnanensis*.

The ratio of three needle clusters for artificial hybrids and three other species are shown in Figure 3. The ratio of three needle clusters in *P. tabuliformis* was 3.33%, while it was 46.67% in *P. yunnanensis*. In addition, the ratio was 26.09% in *P. densata* and 22.54% in artificial hybrids. The ratios of three needle clusters in both *P. densata* and artificial hybrids were between the two parental species.

The mean and standard deviations of each needle trait for artificial hybrids and the three pine species are shown in Table 5. Nineteen needle traits in *P. tabuliformis* were greater than those of *P. yunnanensis*, while only NL and four needle area ratio traits were less than those of *P. yunnanensis*. The majority of needle traits in artificial hybrids and *P. densata* (15 and 17, respectively) were between *P. tabuliformis* and *P. yunnanensis*.

**Table 5:** Statistics of measured needle traits for artificial hybrids and the species tested

Traits (unit)	Artificial hybrid	*Pinus tabuliformis*	*P. densata*	*P. yunnanensis*	The performance of artificial hybrid	The performance of *P. densata*
NL (mm)	88.02 ± 32.73^b^	64.47 ± 12.81^a^	88.78 ± 22.49^b^	97 ± 25.73^b^	*P.y*-like	*P.y*-like
NW (mm)	1.17 ± 0.16^b^	1.25 ± 0.13^b^	1.03 ± 0.15^a^	0.98 ± 0.05^a^	*P.t*-like	*P.y*-like
NT (mm)	0.68 ± 0.11^b^	0.67 ± 0.07^b^	0.64 ± 0.07^a^	0.58 ± 0.06^a^	*P.t*-like	*P.y*-like
CSRN (No.)	6.53 ± 1.53^b^	6.78 ± 1.17^b^	6.03 ± 0.86^b^	4.67 ± 1.18^a^	*P.t*-like	*P.t*-like
FSRN (No.)	5.65 ± 1.23^b^	5.66 ± 1.00^b^	4.17 ± 0.90^a^	4.07 ± 0.72^a^	*P.t*-like	*P.y*-like
CSR2N (No.)	20.78 ± 2.21^a^	21.83 ± 1.89^a^	20.54 ± 2.05^a^	21.67 ± 1.18^a^	*P.t/P.y*-like	*P.t/P.y*-like
FSR2N (No.)	20.33 ± 2.15^a^	21.59 ± 2.07^a^	20.54 ± 1.87^a^	21.33 ± 1.63^a^	*P.t/P.y*-like	*P.t/P.y*-like
CSD (No./mm^2^)	58.78 ± 16.21^a^	59.99 ± 13.5^a^	61.52 ± 14.49^a^	52.08 ± 14.37^a^	*P.t/P.y*-like	*P.t/P.y*-like
FSD (No./mm^2^)	49.56 ± 12.57^a^	49.24 ± 9.71^a^	42.21 ± 9.92^a^	44.6 ± 8.54^a^	*P.t*/*P.y*-like	*P.t/P.y*-like
MSD (No./mm^2^)	54.17 ± 12.99^a^	54.61 ± 10.31^a^	51.87 ± 10.43^a^	48.34 ± 6.43^a^	*P.t/P.y*-like	*P.t/P.y*-like
VBW (mm)	0.51 ± 0.08^c^	0.53 ± 0.07^c^	0.45 ± 0.06^b^	0.37 ± 0.02^a^	*P.t*-like	Extreme (−)
VBT (mm)	0.30 ± 0.05^b^	0.29 ± 0.04^b^	0.29 ± 0.03^b^	0.24 ± 0.03^a^	*P.t*-like	Extreme (−)
NSA (mm^2^)	0.60 ± 0.15^b^	0.65 ± 0.12^c^	0.50 ± 0.09^b^	0.38 ± 0.06^a^	Intermediate	Intermediate
NSP (mm)	3.10 ± 0.38^c^	3.28 ± 0.32^c^	2.79 ± 0.30^b^	2.49 ± 0.18^a^	*P.t*-like	Extreme (−)
VBA (mm^2^)	0.13 ± 0.04^b^	0.13 ± 0.03^b^	0.11 ± 0.02^b^	0.07 ± 0.01^a^	*P.t*-like	Extreme (−)
VBP (mm)	1.34 ± 0.21^bc^	1.38 ± 0.17^c^	1.21 ± 0.15^b^	1.01 ± 0.05^a^	*P.t*-like	Extreme (−)
RCN (No.)	3.69 ± 1.66^b^	3.80 ± 1.42^b^	3.65 ± 1.52^b^	2.20 ± 0.45^a^	*P.t*-like	Extreme (−)
RCA (mm^2^)	0.002 ± 0.00^ab^	0.002 ± 0.00^b^	0.002 ± 0.00^ab^	0.001 ± 0^a^	*P.t*/*P.y*-like	*P.t*/*P.y*-like
RCP (mm)	0.32 ± 0.16^b^	0.35 ± 0.13^b^	0.30 ± 0.12^ab^	0.20 ± 0.04^a^	*P.t*-like	*P.t*/*P.y*-like
MA (mm^2^)	0.47 ± 0.12^bc^	0.52 ± 0.10^c^	0.39 ± 0.07^b^	0.31 ± 0.05^a^	*P.t*-like	Intermediate
MA/VBA	3.77 ± 0.64^a^	4.09 ± 0.56^a^	3.76 ± 0.43^a^	4.19 ± 0.60^a^	*P.t*/*P.y*-like	*P.t*/*P.y*-like
MA/RCA	311.8 ± 126.8^a^	262.6 ± 75.6^a^	292.4 ± 158.6^a^	294.6 ± 114.3^a^	*P.t*/*P.y*-like	*P.t*/*P.y*-like
MA/(VBA + RCA)	3.72 ± 0.62^a^	4.02 ± 0.55^a^	3.70 ± 0.41^a^	4.13 ± 0.60^a^	*P.t*/*P.y*-like	*P.t*/*P.y*-like
VBA/RCA	84.53 ± 36.52^a^	65.70 ± 21.48^a^	78.76 ± 45.32^a^	68.98 ± 20.72^a^	*P.t*/*P.y*-like	*P.t*/*P.y*-like

MSD, mean stomatal density; NL, needle length; NSA, needle section area; NSP, needle section perimeter; NT, needle thickness; NW, needle width; RCA, resin canal area; RCN, resin canal number; RCP, resin canal perimeter; VBA, vascular bundle area; VBP, vascular bundle perimeter; VBT, vascular bundle thickness; VBW, vascular bundle width.

Mean with different superscript letters (a, b, c) are significantly different (*P* < 0.05) as determined by Duncan multiple range tests. Data in the table: mean ± SD. The traits are positively (+) or negatively (−) extreme where the values for artificial hybrid and *P. densata* are significantly higher or lower than those of its parental species; intermediate where the traits for artificial hybrid and *P. densata* are significantly different from but intermediate between those of the parental species; traits that do not differ significantly from one or both parental species are designated *P.t*-like, *P.y*-like, or *P.t/P.y*-like.

The results of ANOVA on 24 needle traits between artificial hybrids and three other species are shown in Table 6. Significant (*P* < 0.05) variations were observed among species for 22 needle traits, excluding CSD and MSD. Variations for individuals within species (or artificial hybrids) were significant for all needle traits. The variance components of the 22 needle traits among species ranged from 2.01% to 25.68%. The broad heritabilities of three species and artificial hybrids were moderate or higher (ranging from 0.43 to 0.93) for 22 needle traits.

**Table 6:** ANOVA of needle traits among artificial hybrids and the three pine species

Traits^1^	Mean square			Variance component (%)			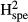	R_ind_
	
	Species^2^	Ind (spe)^3^	Error^4^	Species	Ind (spe)	Error		
NL	19134.250**	2267.251**	21.222	19.31	78.47	2.22	0.88	0.99
NW	0.899**	0.059**	0.010	25.68	45.18	29.14	0.93	0.82
NT	0.088**	0.028**	0.004	5.12	62.37	32.51	0.68	0.85
CSRN	16.246**	4.821**	1.184	4.95	48.07	46.98	0.70	0.75
FSRN	49.138**	3.451**	0.758	23.16	41.65	35.18	0.93	0.78
CSR2N	27.392**	10.937**	3.470	2.93	40.54	56.53	0.60	0.68
FSR2N	22.963**	8.564**	3.560	2.92	30.97	66.11	0.63	0.59
CSD	337.250	550.435**	163.983	0.0	43.99	56.01	0.00	0.70
FSD	819.032**	330.522**	96.652	2.97	43.32	53.71	0.60	0.71
MSD	55.482	357.259**	81.293	0.0	53.09	46.91	0.00	0.77
VBW	0.162**	0.015**	0.003	19.07	46.39	34.53	0.91	0.80
VBT	0.023**	0.006**	0.001	7.19	62.39	30.42	0.74	0.86
NSA	0.512**	0.059**	0.004	17.88	65.99	16.13	0.88	0.93
NSP	4.863**	0.372**	0.035	24.95	57.10	17.95	0.92	0.91
VBA	0.023**	0.004**	0.000	12.87	67.16	19.97	0.83	0.91
VBP	0.842**	0.093**	0.013	17.07	55.83	27.10	0.89	0.86
RCN	13.243**	6.384**	1.015	2.60	62.14	35.25	0.52	0.84
RCA	0.0061**	0.0035**	0.0003	2.01	74.57	23.42	0.43	0.91
RCP	0.124**	0.063**	0.007	2.52	69.93	27.55	0.49	0.88
MA	0.327**	0.036**	0.003	18.75	65.37	15.88	0.89	0.93
MA/VBA	3.233**	0.964**	0.126	5.76	65.02	29.21	0.70	0.87
MA/RCA	50168.78**	55252.88**	10484.25	0.0	58.73	41.27	0.00	0.81
MA/(VBA + RCA)	3.029**	0.911**	0.120	5.69	64.86	29.45	0.70	0.87
VBA/RCA	8916.711**	4599.377**	819.176	2.22	59.26	38.52	0.48	0.82

We abbreviate individual and species as ind and spe, respectively.

*P < 0.05; **P < 0.01 for all variables in species and ind (spe).

1 See Table 3 for definitions of the variables.

2 df = 3 for species.

3 df = 193 for individuals within species.

4 df = 394 for error.

Thirteen traits in artificial hybrids differed significantly from *P. yunnanensis*, and only NL and NSA were significantly different from *P. tabuliformis*, indicating that the maternal effect had a greater impact on needle traits in artificial hybrids. A total of 19 needle traits did not differ significantly between artificial hybrids and *P. densata*, which suggested that the artificial hybrids had similar needle features as *P. densata*.

Nine needle traits in *P. densata* differed significantly from those in *P. tabuliformis* and *P. yunnanensis*, and five traits differed significantly among the three pine species. The needle anatomical traits examined in this study showed significant differences among the three species. Therefore, needle anatomical traits could be used as taxonomic references for identification.

The 24 needle traits in artificial hybrids could be divided into three groups (Table 5) based on different significance levels between the two parental species according to Duncan's multiple range tests: intermediate, *P. tabuliformis* -like, or *P. yunnanensis* -like (Ma et al. 2010).

**Figure 4:**
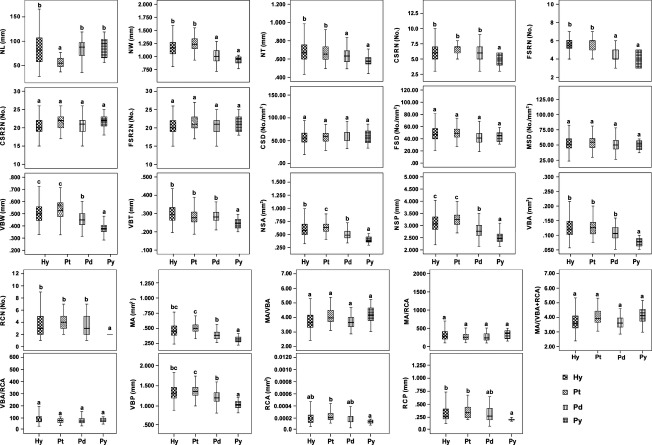
Box plots of the needle traits in artificial hybrids and three pine species. Boxes represent the interquartile range encompassing 50% of the values. The bar within each box indicates the median. Hy: artificial hybrid, Pt: *Pinus tabuliformis*, Pd: *P. densata* and Py: *P. yunnanensis*. Different lowercase (a–c) shows significantly different (*P* < 0.05) between artificial hybrids and three species.

The medians, quartiles, and ranges of the needle phenotype traits in each of the pine samples are summarized in the box plot (Fig. 4). The range of most traits was large in artificial hybrids, spanning nearly the complete numerical range of the traits in the two parental species and *P. densata*.

### Comparison between hybrid families with the three pine species

**Figure 5:**
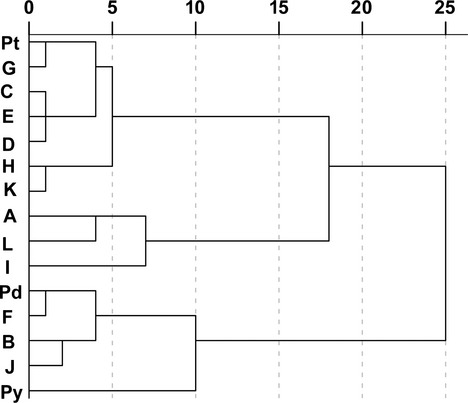
Cluster analysis of the 12 hybrid families and the three pine species based on needle morphology and anatomy traits. A–L: 12 artificial hybrid families, Pt: *Pinus tabuliformis*, Pd: *P. densata*, Py: *P. yunnanensis*.

As genetic variation among different parental combinations may be a source of novel species, 12 artificial hybrid families were compared with maternal and paternal species, as well as *P. densata*. Based on the dendrogram (Fig. 5), the three pine species and 12 hybrid families could be divided into four categories, in which hybrid families I, A, and L were clustered into one group. The hybrid families B, F, and J among the most simulated hybrid families with *P. densata* were clustered into another group. The other hybrid families and *P. tabuliformis* clustered into the third group, while *P. yunnanensis* alone formed the fourth group. The hybrid families that clustered into one category mostly had the same female or male parent (Table 1 and Fig. 5). Therefore, parental tree effects may impact HF features. More hybrid families had the same female parent than the same male parent, which indicated that the maternal effect was stronger than the paternal effect.

**Figure 6:**
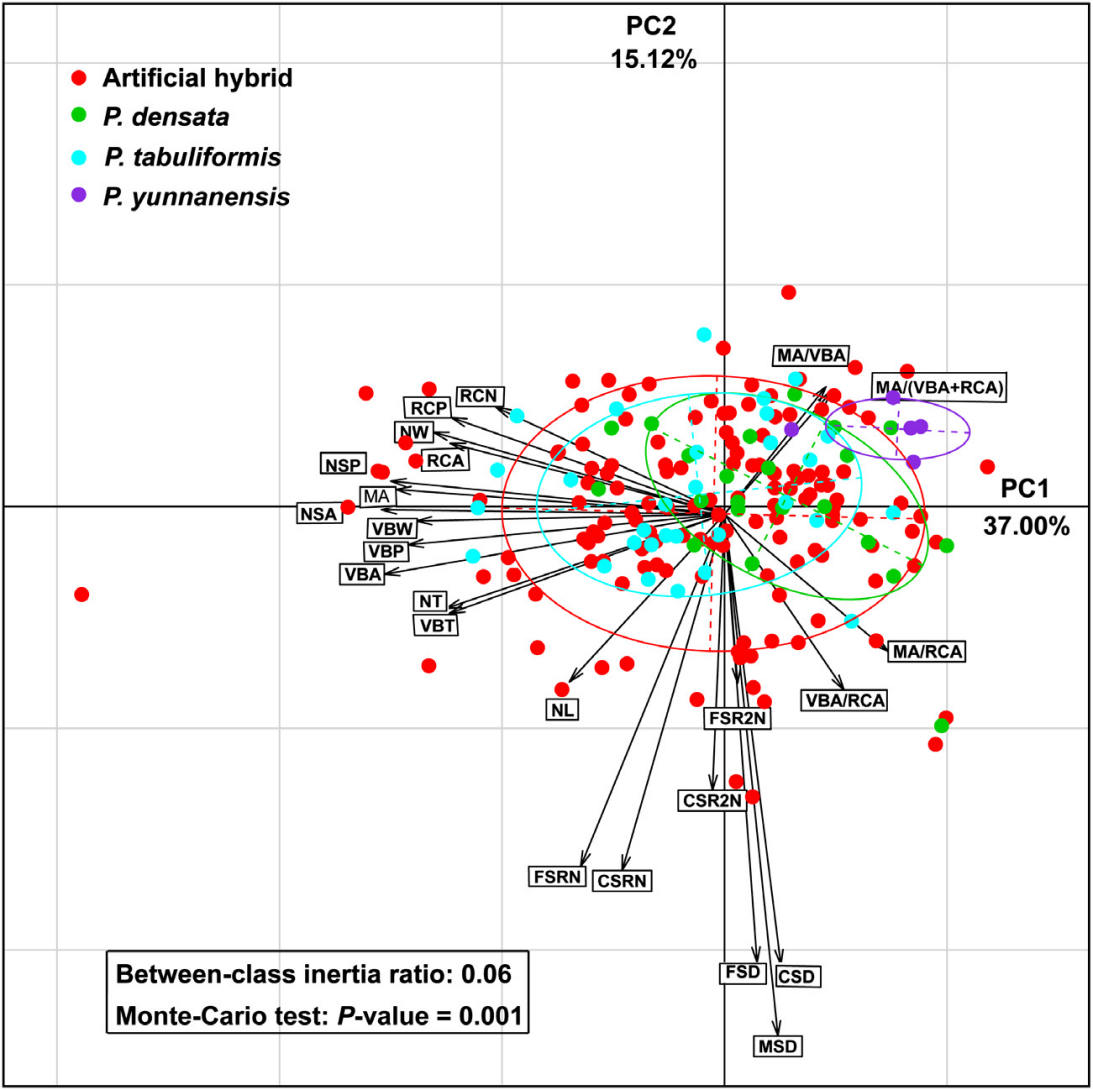
Distance biplot of needle traits based on the principal components analysis (PCA) for the artificial hybrid and the three pine species. Refer to Table 2 for definitions of the traits.

Principal components analysis of the 24 needle traits identified two components with eigenvalues greater than 1.0, which collectively explained 52.12% of the observed variation in the needle morphological and anatomical traits, accounting for 37.00% and 15.12%, respectively, of the total variation (Fig. 6). The PCA distance biplot illustrated the relative contribution of each needle variable to PC1 and PC2. Component PC1 was closely associated with needle anatomical traits, and PC2 was associated with needle morphological traits, while the four area ratio traits between different organizational structures in needle cross sections on PCA biplots played an important role in the identification of individuals. According to the reduced dimensionality of the needle phenotype, artificial hybrids showed a broad distribution that completely overlapped with *P. tabuliformis* and *P. densata* and partly overlapped with *P. yunnanensis*. In addition, *P. tabuliformis* and *P. yunnanensis* were separated. *P. densata* overlapped with *P. tabuliformis* and was separated from *P. yunnanensis*.

These results illustrated the large variability in morphological and anatomical needle traits in artificial hybrids, which overlapped with all parental species and *P. densata*, and were consistent with Duncan's multiple range tests and box-plot analysis.

Unfortunately, poor survival in the second and third years of the seedlings that were a reciprocal hybrid of *P. yunnanensis* as maternal and *P. tabuliformis* as paternal meant these were not available. Therefore, there were only seedlings in hybrid of *P. tabuliformis* (maternal) and *P. yunnanensis* (paternal) used in our analyses.

## Discussion

### Similarity of needle characteristics between artificial hybrids and *P. densata*

Needle traits of the artificial hybrids showed different degrees of variation among hybrid families. Parental species effects and individual variation significantly affected the artificial hybrid needle phenotypes, and the variance component was stronger in individuals than in hybrid families. The primary factors affecting individual variability are genetic diversity and microenvironmental effects (White et al. 2007). Among the 12 hybrid families, needle traits in five hybrid families were not significantly different from those of the female parent (*P. tabuliformis*), and needle traits of four hybrid families were not significantly different from *P. densata*. However, all hybrid families were significantly different from the male parent species (*P. yunnanensis*). These results indicated that 12 hybrid families showed high similarity to the female parent species or *P. densata*, but differed from the male parent species. Major et al. (2003) conducted hybridization experiments on red spruce and black spruce and reported that many seedling traits showed a strong maternal effect. The cluster analysis of hybrid families and parental species indicated that the maternal effect was greater than the paternal effect.

In this study, several needle phenotype traits of the hybrid families were higher or lower than the parents, but whether this was caused by heterosis or transgressive segregation remains unclear. Transgressive segregation commonly occurs in the early generations of hybrids, with hybrids exhibiting extreme phenotypic values compared to parental species (Rieseberg et al. 1993, 2003; Burke et al. 1998; Rieseberg and Carney 1998; Buerkle et al. 2000; Ludwig et al. 2004). In a survey of 171 studies that reported phenotypic variation in segregating hybrid populations, Rieseberg et al. (1999) found that 91% reported at least one transgressive trait, and 44% of 1229 traits examined were transgressive. Based on these results, the needle phenotypes may have experienced transgressive segregation.

Although high variability was observed among hybrid families, the mean values of most needle traits in artificial hybrids showed high similarity to *P. densata*. Nineteen needle traits did not differ significantly between artificial hybrids and *P. densata*, which suggested that they may have the same genetic basis.

The ancestral *P. densata* populations originated from hybridization between *P. tabuliformis* and *P. yunnanensis*, of which *P. densata* may be a very large population that experienced complex evolutionary events. Effects such as the frequency of hybridization, backcrossing, founder effect, mating system, and bottleneck may impact the evolutionary history of *P. densata*. This complex genetic polymorphism and linkage disequilibrium pattern indicated that *P. densata* has a broad genetic basis (Gao et al. 2012). Hybridization provides a foundation for natural selection, and large variation in needle traits among hybrid families and individuals may reflect the early generations of *P. densata*. The similarities between artificial hybrids or hybrid families to *P. densata* in needle traits may reflect the early germplasm basis of *P. densata*.

Although molecular genetic analysis confirmed that *P. densata* is a homoploid hybrid of *P. tabuliformis* and *P. yunnanensis*, both species may have acted as the female or male parents, respectively. The present population of *P. densata* has evolved after several million years (Yu et al. 2000; Wang et al. 2001; Song et al. 2002, 2003). Although the experimental population samples of *P. densata* were collected at the westernmost distribution area of the species and distant from the initiation region of natural hybridization (the easternmost distribution area of *P. densata* in western Sichuan; Gao et al. 2012), both population samples of the parent species are distant from the natural hybridization region. Thus, an increased similarity in needle traits between the artificial hybrids and *P. densata* may occur by chance, which could reveal whether *P. densata* arose through natural hybridization of *P. tabuliformis* with *P. yunnanensis* in a species overlap region.

### Needle traits and adaptive speciation of *P. densata* in high-altitude habitats

*Pinus densata* grows on the eastern Tibetan Plateau, where the parental species cannot normally grow and reproduce. The requirements for wet and dry conditions of *P. densata* fall between those of the parental species, and in the Tibetan Plateau extreme environment, many ecological factors such as air temperature, humidity, soil temperature, moisture conditions, and ultraviolet (UV) radiation intensity have changed significantly over the past several million years (Mao and Wang 2011). In the new habitats to which the hybrid has adapted, the parental species are often at a competitive disadvantage and become extinct in the region with an environment suitable for the hybrid (Lexer et al. 2003; Gross et al. 2004; Ludwig et al. 2004; Rieseberg et al. 2007). The successful colonization of a habitat with such extreme ecological properties would require the development of corresponding adaptive traits in *P. densata* that allow it to survive. *Pinus densata* speciation may be related to uplift of the Tibetan Plateau, which altered ecological environmental factors and put pressure on plant morphology. To some extent, the internal organizational structures of the plants correspond to such changes. Such internal structural changes are typically consistent with changes in environmental conditions and are in the direction of adaptation to the environment (Körner et al. 1989; Welch and Rieseberg 2002; Mao and Wang 2011). In the process of plant evolution, needles are sensitive to environmental changes and have a certain degree of plasticity (Zhang et al. 2012). One can determine whether a species is adapted to the environment based on needle growth conditions or morphology and anatomical structures (Gindel 1973).

Differences in the number of needles in a cluster between species and artificial hybrids may influence the size of the needle crossing section, as well as related traits. Thus, we used eight relative traits [CSR2N, FSR2N, MSD, MA/VBA, MA/RCA, MA/(VBA + RCA), VBA/RCA] and NL (distinct from needle size) to compare variations and physiological fitness potential in the environment between species and artificial hybrids. We found that all eight traits were similar between artificial hybrids and *P. densata*, and showed a similar trend with the two parental species. The values of traits such as NL, CSR2N, FSR2N, and MSD fell between those of the parental species, while MA/VBA and MA/(VBA + RCA) were lower than MA/RCA, and VBA/RCA was higher than in the parental species. The area ratio traits reflect interactions among the needle organizational structures.

As an ancient homoploid hybridization-originated species, *P. densata* has experienced systematic and dispersal processing of speciation under high-altitude niche stress based on molecular genetic analysis (Gao et al. 2012), and its needle trait features would experience the same evolutionary pattern. Genetic variations in needle traits between artificial hybrids and the three pine species may be consistent with fitness in their niche. The niche of each pine species differs; *P. densata* grows in higher-altitude valley regions under cooler and wet air conditions, *P. yunnanensis* grows in subtropical regions with much warmer air and abundant rainfall, and *P. tabuliformis* grows in warm temperate zones with a cooler and dry climate (Wu 1956; Guan 1981; Mao and Wang 2011). Compared with the parent species, the relatively higher mesophyll area/VBA ratio and mesophyll area/(resin canals area and VBA) ratio, and the lower mesophyll area/resin canals area ratio and VBA/resin canals area ratio of *P. densata* are the result of needle morphology evolution. The moderate stomata numbers and stomata density (CSR2N, FSR2N, and MSD) in *P. densata* may be the result of adaption to the high-altitude niche. In other words, the developed conducting tissues and the moderate number of gas-exchange organs in needles of *P. densata* may be allow for successful growth in certain regions of the Tibetan Plateau. In addition, plant leaf length may be associated with altitude and temperature conditions (England and Attiwill 2005). The longer NL in both *P. densata* and artificial hybrids than in *P. tabuliformis*, and the shorter length than *P. yunnanensis* were indicative of environmental adaptation. The similar relative needle features and NL in artificial hybrids with *P. densata* may explain the *P. densata* speciation through hybridization and under specific ecological stress.

In summary, significant variations were observed in 10 needle traits among artificial hybrid families and 22 traits among species and artificial hybrids. In addition, variations in all needle traits among individuals within species or artificial hybrids were significant and were under moderate genetic control. Nineteen needle traits in artificial hybrids were generally similar to those in *P. densata* and between the two parental species, *P. tabuliformis* and *P. yunnanensis*. The percentage of plants with three needle clusters in artificial hybrids was 22.92%, which was very similar to *P. densata*. The eight needle traits [NL, CSR2N, FSR2N, MSD, MA/VBA, MA/RCA, MA/(VBA + RCA), VBA/RCA] that were not affected by needle size and would be relative to physiological adaptability were very similar and showed the same patterns in artificial hybrids and *P. densata*. Our results support that *P. densata* arose from homoploid hybridization and speciation in a unique niche.
